# Dingoes at the Doorstep: Home Range Sizes and Activity Patterns of Dingoes and Other Wild Dogs around Urban Areas of North-Eastern Australia

**DOI:** 10.3390/ani6080048

**Published:** 2016-08-16

**Authors:** Alice T. McNeill, Luke K. -P. Leung, Mark S. Goullet, Matthew N. Gentle, Benjamin L. Allen

**Affiliations:** 1 School of Agriculture and Food Sciences, The University of Queensland, Gatton, Queensland 4343, Australia; alice.mcneill@uqconnect.edu.au (A.T.M.); luke.leung@uq.edu.au (L.K.-P.L.); 2 FeralsOut, Kippa-Ring, Queensland 4021, Australia; mark.goullet@feralsout.com; 3 Robert Wicks Pest Animal Research Centre, Biosecurity Queensland, Department of Agriculture and Fisheries, Toowoomba, Queensland 4350, Australia; Matthew.Gentle@daf.qld.gov.au; 4 Institute for Agriculture and the Environment, University of Southern Queensland, Toowoomba, Queensland 4350, Australia

**Keywords:** adaptive kernel, *Canis lupus dingo*, habitat use, human–carnivore conflict, predator management, stray dog, urban ecology

## Abstract

**Simple summary:**

Conflicts with dingoes and other wild dogs are becoming increasingly common in and around urban areas of Australia. A lack of basic information about wild dog movement ecology hampers efficient planning and allocation of resources to mitigate human–wild dog conflicts. We captured, collared and released 37 wild dogs in urban areas of north-eastern Australia to investigate their movement ecology. In general, wild dogs occupied small fragments of bushland within an urban matrix, were active at all times of the day, and lived within a few hundred meters of houses and humans at all times. We conclude that wild dog management strategies in urban areas should focus on the mitigation of impacts at the individual or group level, and not population-level reductions in numbers.

**Abstract:**

Top-predators around the world are becoming increasingly intertwined with humans, sometimes causing conflict and increasing safety risks in urban areas. In Australia, dingoes and dingo × domestic dog hybrids are common in many urban areas, and pose a variety of human health and safety risks. However, data on urban dingo ecology is scant. We GPS-collared 37 dingoes in north-eastern Australia and continuously monitored them each 30 min for 11–394 days. Most dingoes were nocturnal, with an overall mean home range size of 17.47 km^2^. Overall mean daily distance travelled was 6.86 km/day. At all times dingoes were within 1000 m of houses and buildings. Home ranges appeared to be constrained to patches of suitable vegetation fragments within and around human habitation. These data can be used to reallocate dingo management effort towards mitigating actual conflicts between humans and dingoes in urban areas.

## 1. Introduction

Predators are influential components of all natural ecosystems, including highly modified ecosystems characteristic of urban areas. However, the altered availability of resources and risk factors (i.e., danger from humans) can also alter the roles and functions of predators in these ecosystems [1,2]; current knowledge about predator ecology and management in rural or wilderness areas does not always apply to urban contexts. Urbanisation is spreading across many areas and increasing human–wildlife conflicts are often reported (e.g., [3,4,5,6]). A greater understanding of urban predator ecology is important for mitigating human-wildlife conflicts and identifying predator traits or resource requirements that may facilitate improved predator management.

Canids and felids are common mammalian predators in urban ecosystems around the world. Native species include red foxes *Vulpes vulpes* in Europe and coyotes *Canis latrans* or bobcats *Lynx rufus* in North America (e.g., [7,8,9]). However, in many cities, non-native feral cats *Felis catus* and feral or stray dogs *Canis familiaris* are by far the most common predators. Across Australia, feral cats are common in urban areas [10], as are dingoes or dingo × domestic dog hybrids, collectively referred to as ‘wild dogs’ [11,12]. Suggested taxonomic nomenclature for Australian wild dogs includes *Canis lupus dingo*, *Canis lupus familiaris*, *Canis familiaris dingo*, *Canis dingo* and *Canis familiaris*, which are each in common and current usage [13]. Regardless of their debated taxonomic name, the study animals we refer to here are what most people would consider to be ‘dingoes’, irrespective of their genotype or phylogeny (see also [11]). Our study animals are specifically *not* what most people in Australia would consider feral or stray dogs.

Negative impacts of urban wild dogs include predation on threatened fauna and zoonotic disease transmission, as well as economic interests such as predation on livestock and companion animals and the loss of amenity or fouling of recreational areas [12]. Positive impacts may include non-consumptive effects as well as predation on other pest species, such as foxes and feral cats. One of the primary impacts wild dogs pose in urban areas is the risk to human health and safety [14,15,16,17] and associated psychological stress, anxiety or fear of attack [18,19]. Key to investigating wild dog impacts and managing their populations is knowledge of their space use and movement patterns.

Whilst many studies have assessed wild dog home ranges and activity patterns in rural and remote areas (Table 1), very few studies have investigated these in urban areas (e.g., [11,20]). Wild dogs in rural areas have been found to have large home range sizes that can exceed 900 km^2^, but are usually 25–80 km^2^ (Table 1). Mean distances travelled each day by rural wild dogs has been recorded as just under 13 km/day [16] or 9–31 km/day [21]. Allen et al. (2013) [11] reported that urban wild dogs have much smaller home ranges (0.37–100.32 km^2^) but travel similar daily distances (9.83–22.12 km/day) to rural wild dogs. However, that study was limited to just nine predominantly juvenile individuals monitored between 5 and 43 days at one study site. Such temporally restricted data can yield inferences that do not adequately describe actual wildlife behaviour observed over longer timeframes [22]. Thus, data from more wild dogs over a greater period of time is needed to better understand wild dog movements in urban areas.

In this study, we aim to describe home range sizes and activity patterns of wild dogs in urban areas of north-eastern Australia. We use data from 37 animals monitored for 11–394 days at four study sites. Consistent with available preliminary data [11], we hypothesise that urban wild dogs will have small home range sizes and restrict their activity to periods when humans are least active.

## 2. Methods

### 2.1. Ethics Statement

The dingo is considered native wildlife under the *Nature Conservation Act 1992*, and is protected in national parks in Australia. Elsewhere in Queensland dingoes and other wild dogs are declared pest species under the *Land Protection (Pest and Stock Route Management) Act 2002*. Approval to undertake the project was granted by the Department of Agriculture, Forestry and Fisheries Animal Ethics Committee (AEC permit number: CA 2013/01/660), and the project was conducted in accordance with this approval. On all occasions, access to study sites and capture locations was granted by the owner/manager of the land (e.g., local or state government authorities, or private land owners) before trapping commenced.

### 2.2. Study Area

The study was undertaken in urban areas of coastal Queensland in north-eastern Australia, in the state of Queensland, and in the municipal areas of City of Gold Coast (centred at: −27.77, 153.24), Moreton Bay Regional Council (centred at: −27.24, 153.03), Sunshine Coast Regional Council (centred at: −26.59, 152.98) and Townsville Regional Council (centred at: −19.26, 146.80) (Figures S1–S4). The former three are located in southeast Queensland, which has an average annual rainfall of 900 mm and mean daily maximum temperature of 27 °C and minimum of 18 °C (www.bom.gov.au). The area is sub-tropical, with bushland fragments characterised by eucalypt woodlands or rainforest with a dense understory of shrubs, notably the invasive *Lantana camara*. Townsville is located in north Queensland, and has a dry tropical climate with an average annual rainfall of 1200 mm, with mean daily temperature maximums of 30 °C and minimums of 21 °C. Bushland fragments are characterised by open woodlands with a grassland understory, commonly referred to as tropical savannah. Additional description of the study sites is available in Allen et al. (2013) [11].

### 2.3. Wild Dog Capture and Tracking

Wild dog capture and collaring was conducted between May 2013 and March 2016 by local and state government animal research and management staff, and contractors. Soft-catch foot-hold traps (Jake traps, Bridgers and Victor #3s) were used to capture wild dogs at the three sites in southeast Queensland, and Collarum^®^ trap devices were used in Townsville. A variety of lures (e.g., dog or fox urine) were placed around traps to attract wild dogs, which were checked at least once daily in the early morning. Captured wild dogs were restrained with a catch pole before attaching a muzzle restraint, being careful to allow breathing during the procedure. We then recorded standard weights and measures (e.g., age, weight, body condition score, sex etc.) before fitting Iridium-linked GPS collars with VHF functionality (Sirtrack, New Zealand) and releasing the wild dog at the point of capture. Collars were programmed to record GPS points every 30 min continuously. All data were remote downloaded to desktop computer via Iridium (satellite) linkage. Collars dropped off the animals at a designated time or when the battery was low.

### 2.4. GPS Data Screening and Filtering

GPS data was screened and filtered before analyses. Occasional collar malfunction meant that GPS points were sometimes obtained at variable intervals with obviously inaccurate geographic locations (e.g., GPS points collected at 1 min intervals with tens of kilometres between sequential points). Hence, GPS points taken at intervals between 25 and 35 min, 55 and 65 min, 85 and 95 min and 115 and 125 min were accepted, and all others were discarded. This process essentially only retained points that were obtained according to the programmed 30 min duty cycle, excluding those that were obtained outside the cycle or were too infrequent for meaningful movement analyses (i.e., those >120 min between sequential points). Positional accuracy of filtered GPS points was assessed using the horizontal dilution of precision (HDOP) values (range 1–15) recorded with each GPS point, with a lower value indicating a more accurate GPS location. Ground-truthing and results of fixed collar tests were consistent with previous studies (e.g., [16,27], see also [28]), with mean HDOP values ranging between 1.26 and 2.17 (Table 2), or <50 m on-ground error. Hence, all filtered GPS points were considered suitable for analyses and none were excluded based on HDOP values. Less than 3% of all GPS data obtained were excluded by this screening and filtering process.

### 2.5. Home Range Size and Activity Patterns

Home range estimation was undertaken in ArcView v9.3 and v10.2 (ESRI Inc., Redlands, CA, USA) using the extensions XToolsPro v7.0 [29], Hawths Tools v3 [30] and Home Range Tools v3 [31]. Although a wide variety of home range estimators are available [32,33,34,35], we chose to use 90% adaptive kernels (AK; *h* = 1) over other techniques due to their widespread use, to retain consistency with previously published work, and because it tends to yield slight overestimates of home range size compared to more recent and robust approaches, thus being a more conservative approach given our aims and hypotheses. To investigate changes in the daily activity periods of dingoes, we plotted the overall mean speed of travel (m/h) for each hour of the day for each wild dog. This approach estimates straight line or point-to-point data and, as such, underestimates the true distance animals are travelling [36]. Conversion of “distance travelled in each hour” or “sum of line lengths in each hour” into speed (m/h) was necessary to standardise across individual datasets which included variable time gaps between sequential GPS points (i.e., to account for the occasional missing data in the programmed 30 min duty cycle). Hence, we here consider speed as a measure of relative activity between individuals, used to indicate when activity peaks and troughs occur and not necessarily a measure of true speed or distance travelled. Two-tailed t-tests were used to assess differences in mean activity between sexes.

## 3. Results

A total of 37 wild dogs (24 female, 13 male) were trapped and collared during the study period (Table 2). Mean age was 27 months (range 5–60). Mean body weight was 16.7 kg (range 13.0–25.0 kg). Mean body condition score was 3.5 (range 2.0–4.5; maximum possible score = 5). Wild dogs were monitored for an average of 153 days (range 11–394 days), yielding a total of 209,551 useable GPS points (Table 2). Wild dog collaring and release procedures took an average of 23 min. The most serious capture injuries observed were swelling of the trapped foot and/or minor cuts and abrasions. Three wild dogs (GCDog04, NBDog02, SCDog11) died or slipped their collar off within 20 days of release, yielded few data, and were therefore excluded from further analysis. Hence, we analysed data from 34 wild dogs.

Overall mean home range size was 17.47 km^2^ (range 0.53 km^2^ for NBDog01 to 66.02 km^2^ for SCDog10; Table 2, Figures S1–S4), and did not differ between males and females (*t* = −0.26, df 26, *p* = 0.80). Linear regression could not demonstrate a relationship between age and home range size (*r* = 0.23, df 34, *p* = 0.19), although mean home range sizes were different between animals 12 months of age and younger and those that were older (*t* = −2.44, df 20, *p* = 0.02). Three wild dogs dispersed during their monitoring periods (GCDog01, GCDog03, SCDog11), so home range calculations for these animals excluded GPS data collected during their dispersal periods. For example, the first home range for GCDog01 was 12.17 km^2^ in size and was situated adjacent to the Gold Coast suburb of Ormeau, and after dispersing approximately 25 km, his second home range was 10.13 km^2^ in size and situated near the suburb of Nerang (Figure S1). The home ranges of each wild dog was situated within or adjacent to built-up urban areas, and at all times wild dogs were within 1000 m of houses and buildings (Figures S1–S4).

**Figure 1:**
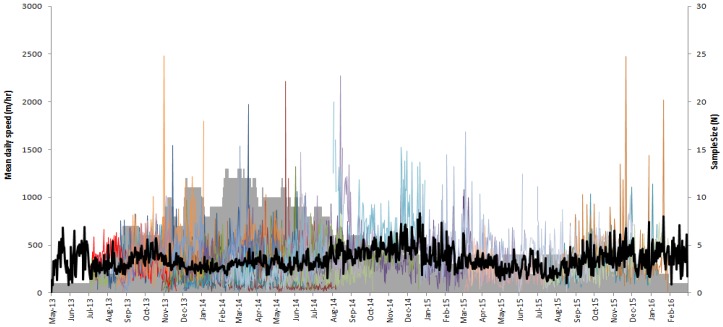
Mean daily activity levels for 34 urban wild dogs monitored in urban areas of north-eastern Australia, between May 2013 and March 2016. Solid line indicates the overall mean, grey area represents sample size.

**Figure 2:**
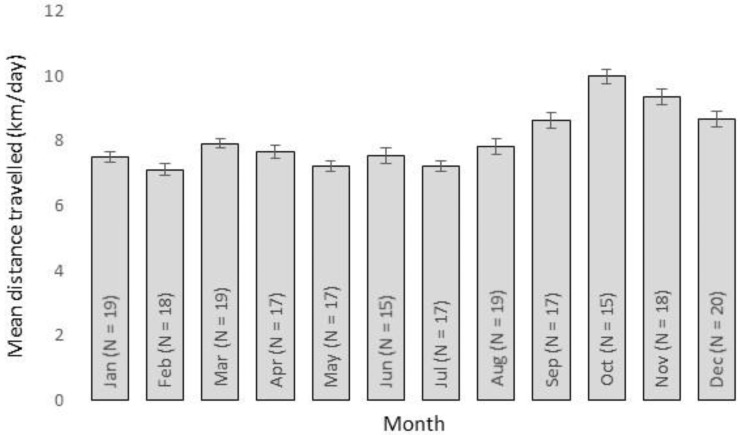
Mean monthly activity levels (SE) for 34 wild dogs monitored in urban areas of north-eastern Australia, between May 2013 and March 2016.

**Figure 3:**
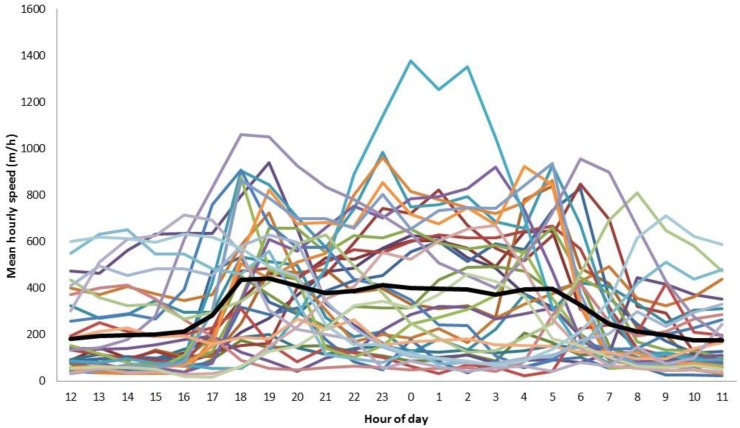
Daily activity patterns for 34 wild dogs monitored in urban areas of north-eastern Australia between May 2013 and March 2016. Solid line represents the overall mean, and coloured lines represent individual wild dogs.

The overall mean daily distance travelled by wild dogs was 6.86 km (range 2.12–13.19 km; Table 2) and did not differ (*t* = −0.22, df 26, *p* = 0.83) between males (mean 6.67 km/day) and females (mean 6.45 km/day). Day-to-day activity patterns fluctuated over time (Figure 1), with a detectable activity peak in October (Figure 2). Individual wild dogs also exhibited highly variable daily activity patterns (Figure 3); four were diurnal (SCDog02, SCDog08, SCDog14, TCDog04), nine were crepuscular (GCDog11, GCDog12, GCDog13, NBDog01, SCDog01, SCDog03, SCDog09, SCDog16, TCDog03), three exhibited no clear pattern (GCDog02, GCDog05, SCDog15) and the remainder were nocturnal, increasing their activity around 18:00 and reducing their activity by 05:00. Sympatric wild dogs living within the same home range exhibited different daily activity patterns.

The type or shape of wild dog home ranges also appeared to vary considerably. Most home ranges were polygonal in shape, exhibiting what might be considered a “normal” home range for wild dogs (Figures S1–S4; [25]). However, SCDog05 occupied a linear home range almost exclusively located along the Bruce Highway (Figure S3). A collared alpha pair of wild dogs and one of their offspring at Townsville (TCDog01, TCDog02, TCDog03) occupied a home range on a large rocky hill completely surrounded by housing and other built-up areas (Figure S4). Wild dogs in north Brisbane likewise had home ranges completely surrounded by housing, with few potential routes for dispersal (Figure S2).

## 4. Discussion

Empirical data on urban wild dog ecology in Australia is scant, and preliminary data on urban wild dog movements previously reported that home range sizes were very small [11]. However, the results of that study were limited by the small sample size and temporal scale, as well as a bias towards juvenile animals. In contrast, our data are derived from predominantly adult animals, from four study sites, and monitored for much greater lengths of time (up to 394 days). Nevertheless, our results support earlier work and demonstrate that urban wild dog home range sizes are indeed smaller than those in rural areas (Table 1 and Table 2, Figures S1–S4). Moreover, the distance travelled per day appeared less for urban wild dogs than their rural counterparts. Variable home range types or shapes suggest behavioural plasticity and flexible resource requirements of urban wild dogs.

Similar to other urban carnivores around the world such as coyotes and foxes [1,2] home range sizes for urban wild dogs (17.47 km^2^, Table 2) appear smaller than those of rural wild dogs, which can exceed 900 km^2^ (Table 1). The largest home range sizes of the wild dogs we monitored were from animals living on the edge of urban areas in wilderness zones within the Gold Coast hinterland, where human disturbance is minimal. In contrast, three of the wild dogs collared in Townsville (excluding TCDog04) had small home range areas (<4 km^2^) on Castle Hill, an isolated fragment of sparse bushland within 200 m of the Townsville central business district (Figure S4). One wild dog, NBDog05, which was collared for 117 days, had one of the smallest home ranges (1.08 km^2^). This animal lived within a small and low-lying patch of *Melaleuca* spp. trees and rainforest alongside a major highway. The spatially restricted movements observed during the relatively long collaring periods indicate that small fragments of bushland in urban areas can provide sufficient resources (e.g., prey, den sites) to sustain wild dogs.

The shapes of these wild dog home ranges appeared to vary considerably across the four study sites, and dispersal behaviour was also observed on a few occasions. Dispersal periods represent times of greater risk to wild dogs as they attempt to secure mates and find a suitable territory which provides for their needs [25]. Of the 37 wild dogs we released, four (i.e., SCDog04, SCDog06, TCDog02, SCDog11) were hit and killed by collision with vehicles during the period we monitored their movements. For example, shortly after release, SCDog11 dispersed and was killed on the Bruce Highway approximately 35 km from his release site. Dispersal in urban areas is likely hazardous to wild dogs due to increased human activity, especially along highways and other main roads.

Our data confirms that wild dogs have flexible spatial requirements and can persist in very small high-risk fragments in urban areas. Highways and roads are one of the major causes of mortality for urban coyotes, with up to 40% of deaths being attributed to road accidents [2]. However, main roads can also provide food sources (i.e., carrion) for scavenging predators. Thus, the occurrence of home ranges adhering closely to such areas (e.g., SCDog05) suggests that some wild dogs are willing to accept the risks of living in such perilous circumstances.

Presumably to mitigate some of the risks associated with urban life (e.g., vehicle collisions), 27 out of the 34 wild dogs we analysed exhibited activity patterns that avoided times of high human activity (Figure 3), similar to what has been found in other urban mammal predators (e.g., [37,38]). Wild dog activity appeared to peak in spring (Figure 2), coinciding with the emergence of pups from dens [25,39] and typical spring increases in prey numbers. Night times are also the periods when activity of wild dog prey (for details, see Allen et al. 2016 [40]) increases, such as macropods and smaller mammals (e.g., [41,42]). In some cases (i.e., animals adjacent to Nambour, Figure S3), wild dogs occupying the same home range area exhibited markedly different activity patterns (Figure 1 and Figure 3), suggesting that intraspecific competition between individuals may also influence wild dog movement and space use. Avoidance of other wild dogs may be one reason why the mean daily distance travelled for urban wild dogs was also relatively high given such small home ranges (Table 2), despite being approximately half the distance travelled by wild dogs in rural areas (e.g., [16,21,43]). However, our coarse analyses of daily activity patterns simply attempted to describe the general pattern of urban wild dog activity, possibly producing ‘noise’ where there may not actually be any noise [44], and future studies of specific diel behaviours should refine this approach.

## 5. Management Conclusions

We have shown that urban wild dogs have relatively small home ranges, variable activity patterns, flexible spatial requirements, and can persist in small fragments of bushland within highly built-up areas. These data have important implications for the management of wild dogs in urban areas. Wild dog management actions are spatially and temporally constrained by human activity in urban areas where it is typically not possible to apply control strategies at spatial scales suitable for reducing wild dog numbers. For example, current control strategies for wild dogs often rely on nil-tenure or cross-tenure attempts at broadscale population-level control using poison baits distributed as densely as possible [12,18]. However, legislation preventing the use of toxins close to residential areas [45] and conflicting attitudes towards wild dog control means that it is very difficult, if not impossible, to reduce urban wild dog populations and impacts using such conventional broadscale strategies designed for rural areas. Control objectives in urban areas may therefore need to focus more on finer scales, or on individuals and groups (not populations) or on specific impacts, to ensure the wild dogs responsible for the impacts are targeted and management resources are not wasted. Our data provides information on space use by such groups and individuals which could be used to reallocate management effort to places and times with a greater chance of successful conflict mitigation. Future research should identify characteristics of wild dog impacts and activity ‘hot spots’ in urban areas and seek to develop and assess suitable tools and strategies that can better mitigate conflicts between humans and wild dogs in these areas. Such research would need to account for potential discrepancies between where wild dogs live and where they cause impact.

## Figures and Tables

**Table 1:** Home range sizes of wild dogs in rural, urban and wilderness areas of northern Australia (adapted from Fleming et al. 2012 [23]). * Exclusive of animals monitored for <20 days.

Ecosystem	Home Range Size km^2^ (SE or Range)	N	Method	Source
Semi-arid tropics	77.3 (22.1)	19	Pooled mean 95% MCP	[24]
Arid	67 (32–126)	5	Not stated	[25]
Monsoonal	39 (15–88)	18	Not stated	[25]
Arid monsoonal	25 (7–110)	24	Not stated	[25]
Arid monsoonal	414.9 (103.5)	9	85% kernel	[26]
Arid	24 (13–32)	7	95% MCP	[27]
Arid	63.5 (16.6–286.3)	7	90% adaptive kernel	[16]
Urban	17.72 (0.37–100.32)	9	90% adaptive kernel	[11]
Urban	17.47 (0.53–66.02)*	37	90% adaptive kernel	Current Study

**Table 2:** Details of urban wild dogs trapped, collared and monitored in north-eastern Australia between May 2013 and March 2016.

Dog ID	Estimated Age	Sex	Weight	Processing Time	Capture Date	Body Condition Score	N = Days Monitored	N = GPS Points Analysed	Mean HDOP	Home Range Size (km^2^)	Mean Daily Distance Travelled (km)
GCDog01	36	M	21.0	23	23-Jul-2013	4.0	248	9268	1.88	12.17/10.13 ^	7.44
GCDog02	16	F	13.7	24	5-Nov-2013	3.0	283	10,644	1.74	4.00	2.12
GCDog03	28	F	17.3	43	7-Nov-2013	4.0	223	8021	1.91	14.59	5.96
GCDog04	28	F	15.0	10	9-Nov-13	3.5	11	175	2.17	7.97	1.20
GCDog05	52	F	19.6	15	10-Nov-13	3.0	225	8921	1.92	9.19	3.01
GCDog06	28	F	14.5	21	14-Nov-2013	4.0	98	2985	1.89	30.94	6.86
GCDog07	34	F	15.0	15	8-Mar-2014	4.0	110	4366	1.68	30.29	8.24
GCDog09	45	M	15.5	15	2-Apr-2014	4.0	110	4247	1.87	45.28	8.93
GCDog10	24	F	25.0	15	25-Jun-2014	3.5	42	1608	2.07	25.69	5.46
GCDog11	60	M	23.0	20	9-Jul-2014	-	255	11,128	1.67	34.52	8.66
GCdog12	24	M	19.0	11	28-Jul-2015	2.0	176	5115	1.50	20.48	5.48
GCdog13	60	F	15.0	16	28-Jul-2015	2.0	192	5412	1.70	21.87	6.72
NBDog01	17	F	16.3	13	10-Dec-2013	4.0	27	1212	1.81	0.53	2.28
NBDog02	17	M	13.0	8	10-Dec-2013	3.0	17	764	1.71	0.26	2.50
NBDog03	29	F	16.5	16	11-Dec-2013	3.5	381	17,465	1.64	4.59	8.79
NBDog04	29	M	21.0	12	12-Dec-2013	4.0	127	5432	1.73	8.21	8.51
NBDog05	29	M	19.7	15	13-Dec-2013	4.5	117	4816	1.76	1.08	3.89
NBDog06	31	M	18.5	9	12-Feb-2014	4.0	99	4468	1.72	16.87	11.21
NBDog07	31	F	15.3	15	14-Feb-2014	3.5	93	4117	1.69	8.95	9.27
SCDog01	12	F	15.3	23	9-May-2013	3.5	203	9608	1.48	7.39	8.01
SCDog02	12	F	13.8	18	10-Jul-2013	3.0	29	1322	1.66	9.41	3.95
SCDog03	25	F	17.8	25	21-Aug-2013	3.0	37	1044	1.92	4.26	3.78
SCDog04	37	M	19.0	16	21-Aug-2013	4.0	116	4913	1.84	13.76	8.76
SCDog05	25	F	14.2	12	21-Aug-2013	3.0	144	5525	1.88	39.73	9.00
SCDog06	42	M	15.7	15	1-Sep-2013	4.0	193	6967	1.80	21.82	9.40
SCDog07	14	F	16.0	16	2-Sep-2013	4.0	81	3752	1.75	13.42	10.36
SCDog08	10	F	21.4	150	18-May-2014	4.0	149	4857	1.83	3.21	4.05
SCDog09	10	F	13.6	14	19-May-2014	4.0	394	16,962	1.26	24.82	9.19
SCDog10	22	F	14.5	16	19-May-2014	4.0	232	9298	1.62	66.02	13.19
SCDog11	24	M	15.0	14	17-Jul-2014	3.0	19	895	1.72	89.64	4.72
SCDog14	5	F	13.4	20	12-Dec-2014	3.0	229	5950	1.73	13.25	6.47
SCDog15	21	M	13.0	45	12-Mar-2015	4.0	156	5710	1.44	31.61	3.68
SCDog16	60	F	18.0	45	17-Jul-2015	4.0	235	9849	1.62	15.89	7.08
TCDog01	30	F	16.0	28	22-Jan-2014	3.0	123	4658	1.72	3.72	4.99
TCDog02	7	M	16.0	28	16-Feb-2014	3.0	226	1560	1.69	3.93	3.53
TCDog03	9	F	13.5	18	18-Mar-2015	3.0	42	1540	1.63	4.09	5.55
TCDog04	21	F	17.5	25	6-May-2015	3.5	219	4956	1.42	35.64	9.37
Total	Mean 27	24F/13M	Mean 16.7	Mean 23		Mean 3.5	Mean 153	Mean 5662	1.73	Mean 17.47 *	Mean 6.86 ^#^

^ Home range sizes before and after dispersal; * Inclusive of both home ranges for GCDog01 and exclusive of animals monitored for <20 days; ^#^ Exclusive of animals monitored for <20 days.

## Supplementary Materials

Supplementary File 1
