# Supplementary material for: Dingoes at the Doorstep: Home Range Sizes and Activity Patterns of Dingoes and Other Wild Dogs around Urban Areas of North-Eastern Australia
Source: Animals (Basel). 2016 Aug 16;6(8):48. doi: 10.3390/ani6080048 (PMC4997273; doi:10.3390/ani6080048)
Supplement: Supplementary File 1 [file animals-06-00048-s001.pdf]

Supplementary Materials: Dingoes at the Doorstep: Home Range Sizes and Activity Patterns of Dingoes and Other Wild Dogs around Urban Areas of North-Eastern Australia

Alice T. McNeill, Luke K. -P. Leung, Mark S. Goullet, Matthew N. Gentle and Benjamin L. Allen

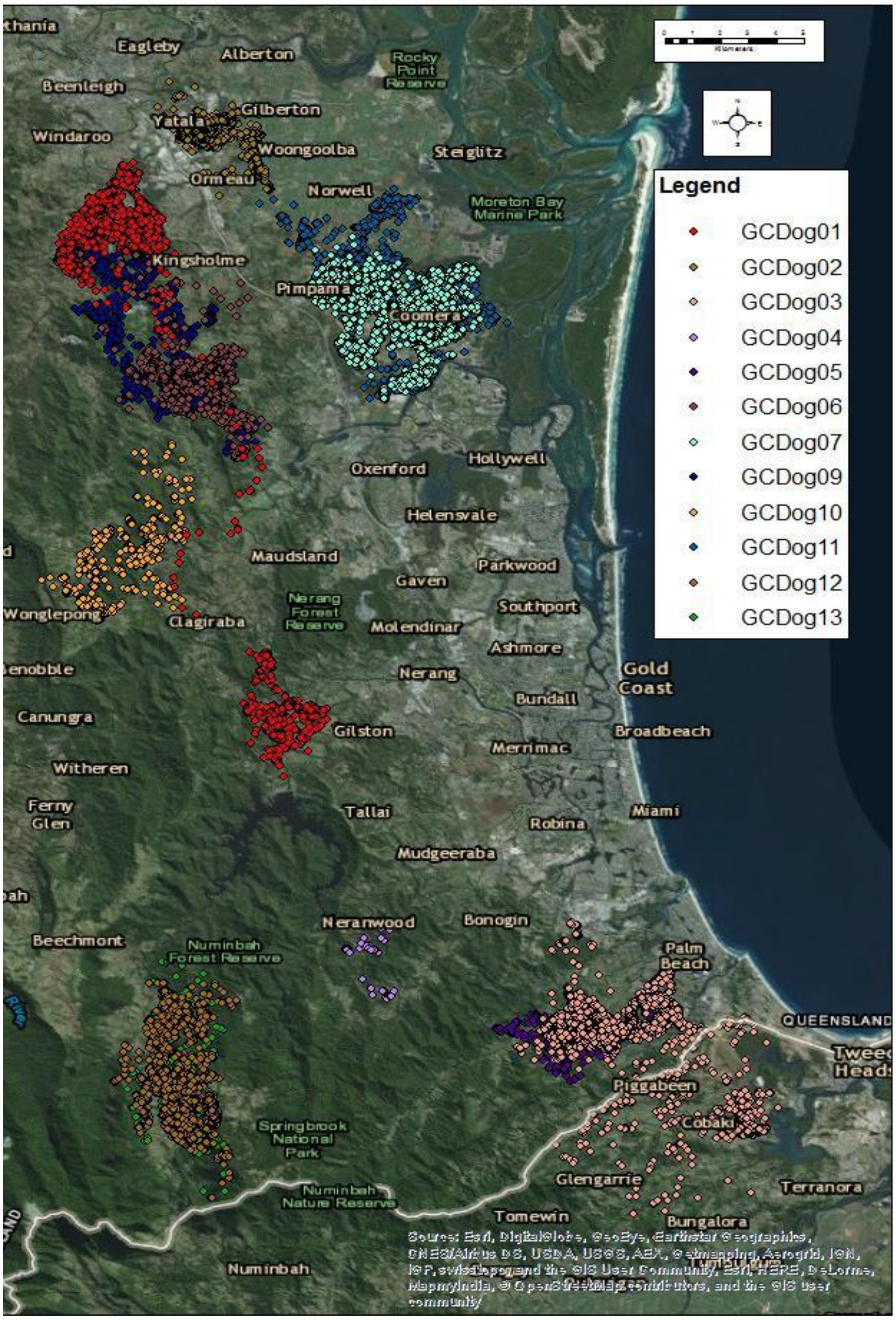

Figure S1. Movements of wild dogs around urban areas of the Gold Coast, Queensland, Australia, July 2013 to February 2016. Coloured dots represent GPS points taken at 30 min intervals, see Table 2 for details.

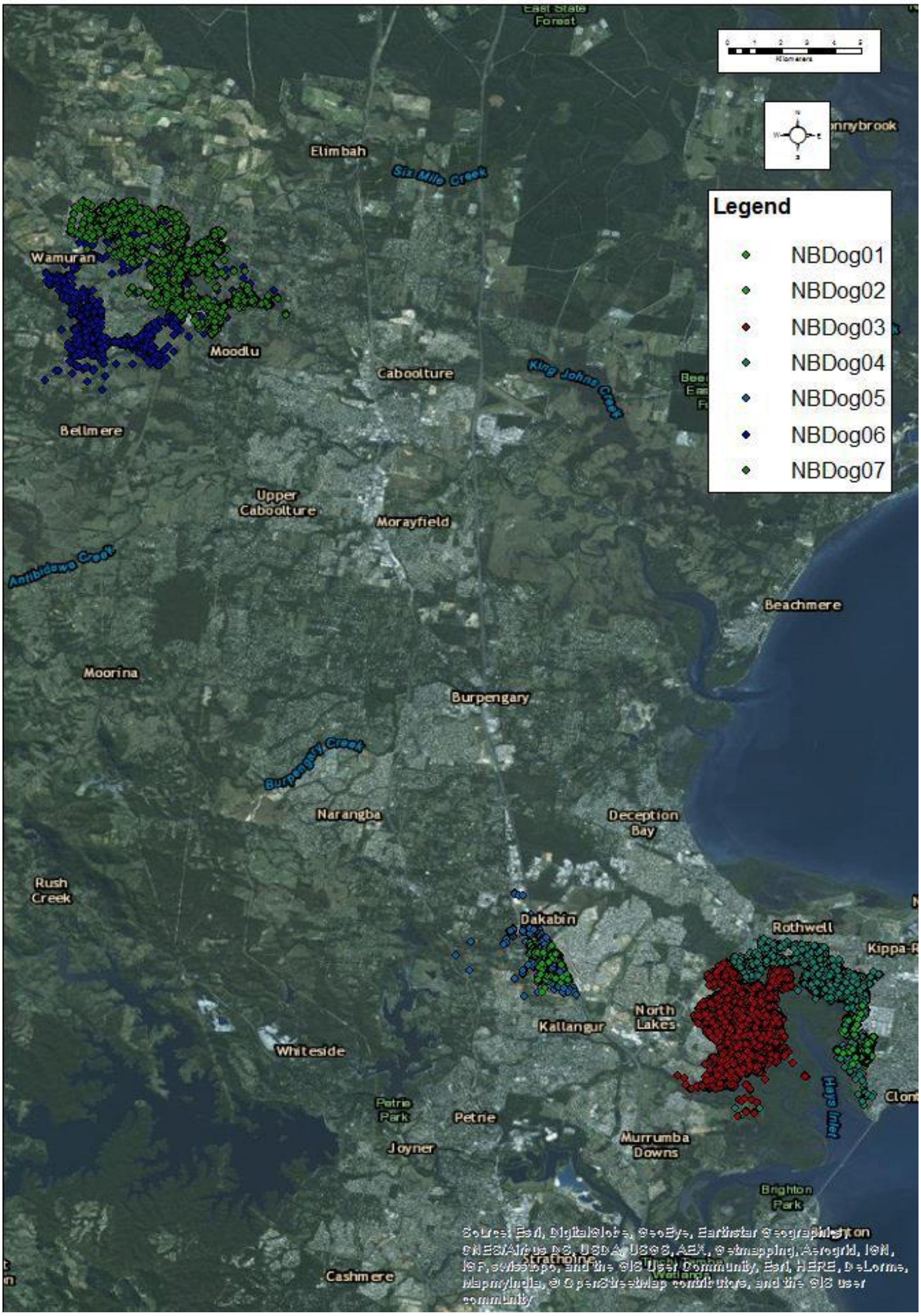

Figure S2. Movements of wild dogs around urban areas of the north Brisbane, Queensland, Australia, December 2013 to May 2014. Coloured dots represent GPS points taken at 30 min intervals, see Table 2 for details.

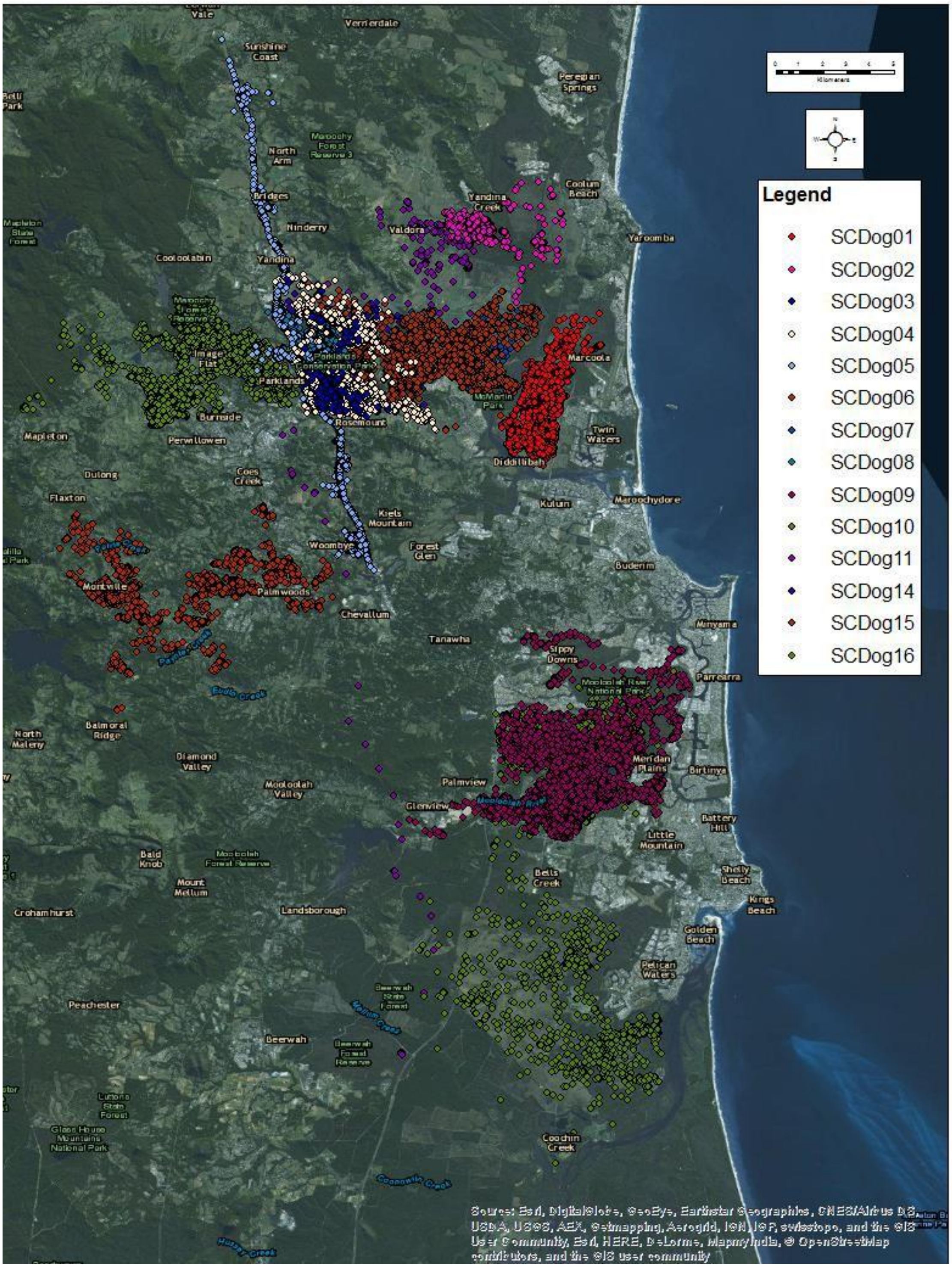

Figure S3. Movements of wild dogs around urban areas of the Sunshine Coast, Queensland, Australia, May 2013 to March 2016. Coloured dots represent GPS points taken at 30 min intervals, see Table 2 for details.

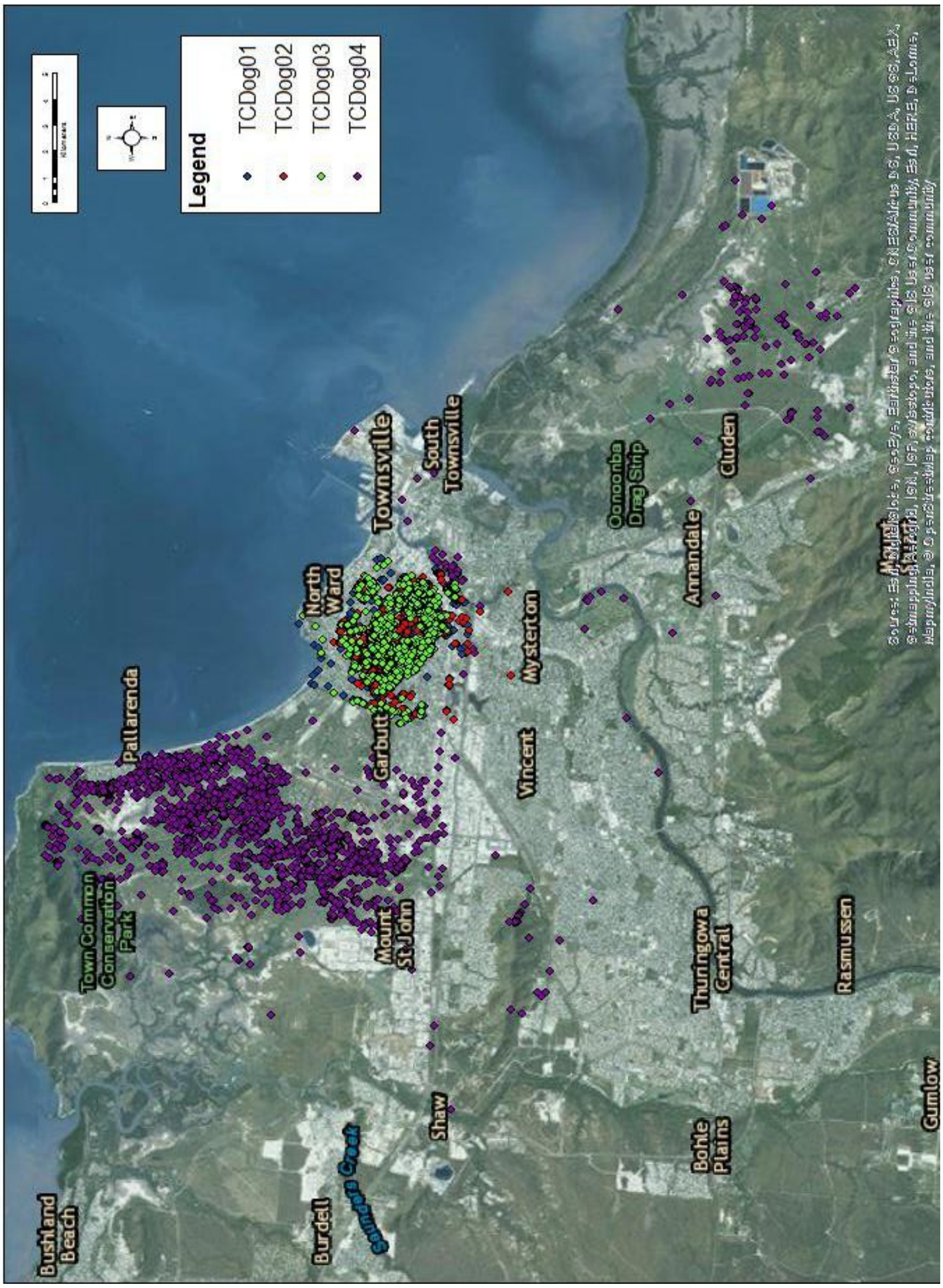

Figure S4. Movements of wild dogs around urban areas of Townsville, Queensland, Australia, January 2014 to December 2015. Coloured dots represent GPS points taken at 30 min intervals, see Table 2 for details.

© 2016 by the authors. Submitted for possible open access publication under the terms and conditions of the Creative Commons Attribution (CC-BY) license (http://creativecommons.org/licenses/by/4.0/).
